# On the Effect of Non-Thermal Atmospheric Pressure Plasma Treatment on the Properties of PET Film

**DOI:** 10.3390/polym15214289

**Published:** 2023-10-31

**Authors:** Irena Maliszewska, Małgorzata Gazińska, Maciej Łojkowski, Emilia Choińska, Daria Nowinski, Tomasz Czapka, Wojciech Święszkowski

**Affiliations:** 1 Department of Organic and Medicinal Chemistry, Faculty of Chemistry, Wrocław University of Science and Technology, 50-370 Wrocław, Poland; daria.kocek@pwr.edu.pl; 2 Department of Polymer Engineering and Technology, Faculty of Chemistry, Wrocław University of Science and Technology, 50-370 Wrocław, Poland; malgorzata.gazinska@pwr.edu.pl; 3 Faculty of Materials Science and Engineering, Warsaw University of Technology, 02-507 Warsaw, Poland; maciek.lojkowski@pw.edu.pl (M.Ł.); emilia.choinska@pw.edu.pl (E.C.); wojciech.swieszkowski@pw.edu.pl (W.Ś.); 4 Centre for Advanced Materials and Technology CEZAMAT, Warsaw University of Technology, 02-822 Warsaw, Poland; 5 Department of Electrical Engineering Fundamentals, Faculty of Electrical Engeenering, Wrocław University of Science and Technology, 50-370 Wrocław, Poland; tomasz.czapka@pwr.edu.pl

**Keywords:** polyethylene terephthalate, non-thermal atmospheric plasma, physicochemical properties

## Abstract

The aim of the work was to investigate the effect of non-thermal plasma treatment of an ultra-thin polyethylene terephthalate (PET) film on changes in its physicochemical properties and biodegradability. Plasma treatment using a dielectric barrier discharge plasma reactor was carried out in air at room temperature and atmospheric pressure twice for 5 and 15 min, respectively. It has been shown that pre-treatment of the PET surface with non-thermal atmospheric plasma leads to changes in the physicochemical properties of this polymer. After plasma modification, the films showed a more developed surface compared to the control samples, which may be related to the surface etching and oxidation processes. After a 5-min plasma exposure, PET films were characterized by the highest wettability, i.e., the contact angle decreased by more than twice compared to the untreated samples. The differential scanning calorimetry analysis revealed the influence of plasma pretreatment on crystallinity content and the melt crystallization behavior of PET after soil degradation. The main novelty of the work is the fact that the combined action of two factors (i.e., physical and biological) led to a reduction in the content of the crystalline phase in the tested polymeric material.

## 1. Introduction

In the past 60 years, polymeric materials (namely, plastics) have become one of the most attractive materials due to their low cost, ease of handling, lightweight, high resistance to physical, mechanical, and chemical ageing, and biological degradation [1,2,3]. The global market size of plastic, which was valued at 579.7 billion USD in 2020, is expected to grow with a compound annual growth rate (CAGR) of 3.2% between 2020 to 2027 [1]. The stability and durability of these materials have been continuously improved, making them resistant to environmental degradation. Initially, its resistance to degradation was considered a beneficial feature. Today, plastic’s durability is recognized as a serious environmental problem [4,5]. Plastic waste has a negative impact on all living organisms, mainly due to its undesirable accumulation in landfills, its leaching into the soil, increased emissions of greenhouse gases, etc. In addition, its influence on aquatic ecosystems is even more damaging as it causes entanglement, ingestion, and intestinal blockage in aquatic animals [4,5,6]. The degradation of synthetic polymers is a slow process that involves several environmental factors.

In general, light irradiation, heat, moisture, and chemical and biological exposure can induce physical and chemical changes in plastic, leading to degradation of the molecular chain. Biodegradation of a polymer material is mainly caused by the activity of various microorganisms and their enzymes [6,7]. Modifications in the physical, optical, and mechanical properties (that is, molecular weight, embrittlement, ductility, chalking, cracking, and color changes) of polymers are directly related to their biodegradation process [2].

Polyethylene terephthalate (PET) is one of the most widely used thermoplastic polymers in the packaging and textile industries. PET production has been estimated to have reached 70 million tonnes in 2020 [3]. It should be noted that more than 10 million tonnes of PET become plastic waste each year [8,9]. Although PET bottles have more than 50% recycling efficiency in the European Union (they are the most recycled plastic product), other PET-based products (i.e., films and fibers) are not recycled efficiently and become a significant source of industrial waste [10].

PET is considered non-biodegradable, but some chemical and physical changes (occurring in natural atmospheric conditions) in the structure of this polymer are observed. Hydrolysis and photolysis are mainly involved in the biodeterioration of PET in nature [4]. Natural weather conditions caused mechanical and chemical fragmentation of PET waste to accumulate over the years, resulting in the formation of PET microplastics (<5 mm), which are even more difficult to track and collect [11,12].

Previously, it was shown that altering the chemical and physical properties of polymeric materials using various techniques, including plasma surface functionalization, plasma deposition, or UV ozonation, increases biodeterioration because the chemically modified polymer is more susceptible to attack by environmental microorganisms [5]. One of these physicochemical treatments can be a non-thermal atmospheric pressure plasma, also called cold plasma.

In general, dielectric barrier discharges (DBDs) are plasmas generated in arrangements with an insulating material (dielectric) between the electrodes [13]. The presence of the dielectric material enables the so-called self-pulsing microdischarges to occur. Moreover, these microdischarges are well-distributed over the dielectric surface; thus, the breakdown is obtained at multiple points instead of at a single point, as in the case of an electric arc. DBDs are a typical example of non-thermal atmospheric discharges or gaseous discharges under normal pressure [14]. Typically, DBDs are driven by high AC voltage in a range up to several tens of kHz [15]. In recent years, an increasing number of studies have been carried out using pulsed or radiofrequency discharges (i.e., frequencies of 1 MHz and above) in systems with DBD-type reactors. In general, the performance of a DBD system significantly relies on the employed process parameters, e.g., gas pressure, gas type, gas flow rate, plasma excitation frequency and power, time, and reactor geometry [16,17,18]. Basic electrical parameters allow not only for control of the discharge; they are also useful for determining discharge modes [19,20]. Currently, there is significant progress in the development of new concepts of high-voltage power supplies for DBD discharge generation, leading to improved homogeneity of the plasma or energy distribution [21,22].

It is important to differentiate between volume DBD and surface DBD [23]. In surface DBD, the electrodes are placed asymmetrically and separated by dielectric barrier discharge, with no additional air gap between the electrodes. Consequently, the discharge occurs at the surface dielectric barrier. The ability to use air in DBD reactors instead of noble gases allows for large-scale use and reduces operating costs [24]. DBD systems have been used for surface treatment, including surface treatment of textiles and polymers [25], ozone synthesis [26], waste gas treatment [27], production of hydrogen [28], water treatment [29], inactivation of microorganisms [30], and lighting and displays [31].

It is well-known that DBD plasma is essentially composed of excited molecules and atoms, positive and negative ions, free radicals, electrons, UV radiation, and reactive oxygen and nitrogen species such as ozone, superoxide, hydroxyl radicals, singlet oxygen, atomic oxygen, nitrogen oxide, or nitrogen dioxide [32].

Treatment of polymeric materials with non-thermal plasma can change the hydrophobicity and roughness of the surface by modifying the adhesion of the surface [7]. It is caused by the generation of polar groups (–COOH and, –OH) and the formation of branched polymer chains [32,33]. Additionally, low molecular weight can form on the plastic surface [34,35]. For example, Gómez-Méndez et al. [36] showed the treatment of low-density polyethylene (LDPE) with a discharge plasma followed by the adhesion, growth, and colonization of *Pleurotus ostreatus*. These authors observed that after 150 days of biodeterioration, the hydrophilicity and roughness of the surface increased. At the same time, *P. ostreatus* showed high lignolytic activities, with the simultaneously high release of a melanin-like pigment type, supporting metabolic stress after exposure to the polymer. Changes of around 27% in the mechanical properties of the LDPE (Young’s modulus and yield strength) after sequential processing supported a higher biodeterioration of the material.

The aim of the work was to examine the effect of two-stage modification (exposition to DBD plasma and soil biodegradation) of a polyethylene terephthalate (PET) film on the properties of this polymer.

## 2. Materials and Methods

### 2.1. Polymeric Film and Chemicals

In our research, a polymeric film made of polyethylene terephthalate (PET) was tested. A PET foil with a thickness of 25 µm was purchased from Adhesive Products Ltd., Waterford, Ireland. All chemical agents were obtained from Avantor Performance Materials Poland S.A., Gliwice, Poland.

### 2.2. Pretreatment of PET Film with Non-Thermal Atmospheric Pressure Plasma

Plasma treatment of the surface of a polymeric material was performed using a dielectric barrier discharge (DBD) plasma reactor operating at atmospheric pressure with air as the working gas. The plasma reactor was powered by a high-voltage power supply with a modulated frequency (Dora PS). The voltage amplitude *U_s_* and the output power *P* were 5.0 kV and 8 W, respectively. The distance between the electrodes was 3 mm. Air at atmospheric pressure was used to generate plasma (no additional external air flow was used). A detailed description of the reactor and the supply voltage source is given in [37]. The polymeric films were cut into square sections with a side length of 30 mm; then, the samples were inserted into a reactor and exposed to the plasma, i.e., only one side of the sample was in direct contact with the plasma. PET samples were treated with plasma for 5 and 15 min. Based on electrical measurements and determination of the efficiency of the plasma source and the size of the area exposed to electrical discharge (cm^2^), the corresponding plasma surface power density was specified as approximately 75 mWcm^−2^. The plasma dose after 1 min of exposure was calculated to be approximately 4.5 Jcm^−2^.

### 2.3. Controlled Biodegradation Test in Soil under Aerobic Conditions

The described test method aims to determine the aerobic biodegradability of plastics. Commercially available garden soil was used in all experiments. The soil was passed through a 2-mm sieve, and total organic carbon (TOC) was measured using the EPA 415.1 method, while total Kjeldahl Nitrogen (TKN) was obtained using the EPA 315.3 method. The soil pH was determined using a soil–water ratio of 1:10 with a glass electrode. The soil was stored in a dark room at 4 °C. Basic chemical and physical properties of the soil are presented in Table S1 (see Supplementary Information).

One hundred grams of the soil sample were transferred to bags and humidity to give 50% WHC and maintained at room temperature for seven days before adding the PET samples. The experimental units were periodically monitored for soil moisture and maintained at an appropriate level of 50% WHC.

PET samples (i.e., 30 mm × 30 mm films) were placed separately in closed jars. Soil acted as both a supporting matrix and a source of soil microorganisms and nutrients. The jars were incubated for 60 days in a dark room at a temperature of 25 ± 0.5 °C, which favors the development of mesophilic soil microorganisms, and under optimal conditions of oxygen and moisture. After the incubation time, the samples were removed from the soil with tweezers and then washed with ethanol and deionized water to remove the microbial biomass and soil residues adhering to the plastic surface. The washed samples were air-dried to a constant weight. Each experiment was carried out in 3 replicates.

### 2.4. AFM Surface Analysis

The morphology of the PET samples and their roughness was evaluated using atomic force microscope MFP 3D BIO (Asylum Research/Oxford Instruments, Santa Barbara, CA, USA) AFM with the ARC 2 controller working in a contact mode.

### 2.5. LFM Surface Mapping

In lateral force microscopy, the AFM tip scans the surface in a direction perpendicular to the longer side of the cantilever. The cantilever bends, respectively, to the friction between the cantilever and the samples surface. The oxidization of the surface layer can increase the friction between the tip and the surface. The cantilever bending is measured as a lateral deflection of the laser beam by the photodetector. The lateral deflection in the primary direction is defined as *Lateral Deflection_trace_* and as *Lateral Deflection_retrace_* when the cantilever scans in opposite direction. The deflection is proportional to the bending measured in millivolts. The friction is measured as follows:

$$Friction=Lateral\;Deflection_{trace}-Lateral\;Deflection_{retrace}.$$

### 2.6. Measurements of Number and Weight Average Molecular Weights

The average molecular weight of the number and weight (*M_n_* and *M_w_*) were determined via a modular-system Agilent 1200 series HPLC with a refractive index detector (RID) equipped with two PLgel 5 µm MIXED-C columns (300 mm × 7.5 mm) in series. Calibration was performed using a set of 8 narrowly distributed polystyrene standards with molecular weight (*M_p_*) in the range of 474–963,000 g/mol.

Measurements were made at 35 °C. HPLC grade chloroform was used as the solvent at a flow rate of 0.7 mL/min. All PET samples were dissolved in hexafluoroisopropanol (HFIP) for 16 h at room temperature. The samples were diluted with chloroform to the final concentration of 2 mg/mL and left for 8 h. Before analysis, samples were filtered through a 0.2 µm PTFE membrane. The data were collected via ChemStation for LC (ver. B.04.02 SP1) and analyzed using ChemStation GPC Data Analysis Software (ver. B01.01).

### 2.7. Fourier Transformed Infrared Spectroscopy (FTIR) Analysis

PET films were analyzed by means of FTIR spectroscopy in the ATR mode. FTIR-ATR spectra were recorded on a Nicolet iZ10 spectrometer (Thermo Scientific, Waltham, MA, USA). Spectra were recorded at the wave number range of 550–4000 cm^−1^, with a spectral resolution of 4 cm^−1^, with 32 coadded scans.

### 2.8. Differential Scanning Calorimetry (DSC) Analysis

DSC measurements were performed using a Mettler Toledo DSC1 system (Columbus, OH, USA), coupled with a Huber TC100 intracooler. The instrument was calibrated using indium (*T_m_* = 156.6 °C, Δ*H_m_* = 28.45 J/g) and zinc (*T_m_* = 419.7 °C, Δ*H_m_* = 107.00 J/g) standards. Samples (~3.5 mg) were measured in 40 µL aluminum plates under a constant nitrogen purge (60 mL/min) from 25 °C to 280 °C. The heating and cooling rate was set to 10 °C/min. The initial degree of crystallinity (*X_c_*) of PET was calculated from the first heating DSC curve according to the following:

$$X_{c}=\frac{\Delta H_{m}}{\Delta H_{m0}^{}}\cdot 100\%,$$

where Δ*H_m_* is melting enthalpy of PET [J/g], and Δ*H*_*m*0_ refers to the equilibrium enthalpy of melting theoretically defined as the enthalpy of melting of 100% crystalline PET, 125 J/g [38].

### 2.9. Wettability Measurements of Polymer Films

Contact angle measurements were performed using a portable computer-based instrument, See System E (Advex Instruments, Brno-Komín, Czech Republic). Before measurement, the samples were cleaned with ethanol. Then, 6 L of distilled water drops of 6 µL were used for the tests. Measurements were carried out in the air under normal conditions of pressure and temperature at a relative humidity of 40%. The test results are given as the arithmetic mean of three independent experiments.

### 2.10. Tests of Dielectric Properties of Polymer Films

To assess the influence of the biodeterioration process on the dielectric properties of PET films, the volume (*ρ_v_*) and the surface resistivity (*ρ_s_*), the dielectric loss factor (tgδ) and electrical strength (*E_f_*) before and after biological exposure, resistance measurements were carried out using the Trek Model 152-1 surface/volume resistance meter (TREK Inc., Lockport, NY, USA) under the IEC 61340-2-3 standard [39]. In our research, the Hameg MH8118 LCR bridge (Rohde and Schwarz, München, Germany) was used to measure the dielectric loss factor according to the standard ASTM D150 standard [40]. The electrical strength was tested according to the IEC 60243-1 standard [41] at a voltage of 50 Hz. The results of individual electrical parameters are given as average values from 6 independent tests.

### 2.11. Formation of Biofilm on PET Film Surface

The soil was extracted in tap water in a ratio of 1:9 by mixing on a shaker for 30 min at 130 rpm. The mixture was then sedimented for a few minutes at room temperature. The soil extract was used as a source of microorganisms that formed a biofilm on the surface of the polymer.

To estimate the efficacy of microbial colonization efficacy on PET samples, the studied materials were cut into 10 mm × 10 mm and placed in 5 mL of soil extract. The samples were incubated for 48 h at room temperature with shaking (130 rpm). After the incubation period, the samples were removed from the soil extract, rinsed twice with distilled water (to remove loosely adherent cells), and placed in a new vessel containing 5 mL of nutrient broth. The biofilm was formed on the surface of the PET sheet for 24 h at room temperature. After this time, the sheet was air-dried for 45 min, placed in a 1% aqueous crystal violet solution (5 mL), and incubated at room temperature for 45 min. Since PET has a lower density than water and floats on its surface, agitation at 130 rpm was used to rinse the entire surface of the sample with the dye. After staining, the sheet was washed five times with sterile distilled water. At this point, the biofilms were visible as purple rings formed on the surface of the PET film. Quantitative analysis of biofilm formation was performed by adding 5 mL of 95% ethanol to decolorize the sheet. The level (OD) of crystal violet present in the decolorizing solution was measured at 595 nm (Shimadzu 1650 UV–vis spectrophotometer (Tokyo, Japan) [42]. Each experiment was carried out in triplicate.

### 2.12. Scanning Electron Microscopy Analysis (SEM)

Plastic samples were cut to 10 × 10 mm and coated with gold nanoparticles as plastics are inherently non-conductive materials. The samples were visualized at different magnifications to analyze the characteristic features after degradation and to study the differences between the control and test samples.

### 2.13. Statistical Analysis

All tests were performed in three repetitions. Statistical analyses were performed using STATISTICA data analysis software (version 13.3, StatSoft Inc., Kraków, Poland) and Excel. Quantitative variables were determined via the arithmetic mean of standard deviation, median, or maximum/min (range), with a 95% confidence interval. The statistical significance of the differences between the two groups was established using Student’s *t* test. In all calculations, a *p*-value of 0.05 was assumed as the limit value.

## 3. Results

Ultrathin PET films have undergone a two-step modification: in the first step, the tested polymer was modified via non-thermal plasma treatment; while in the second step, the plasma-modified polymer was subjected to a 60-day soil biodegradation test (see “Controlled biodegradation test in soil under aerobic conditions”). Experiments on the plasma treatment of PET film were carried out for two treatment times (5 and 15 min). After each modification step, the morphological and physicochemical properties before and after exposure under controlled laboratory conditions were compared. The results obtained are presented below.

### 3.1. Analysis of Surface Morphology

**Figure 1:**
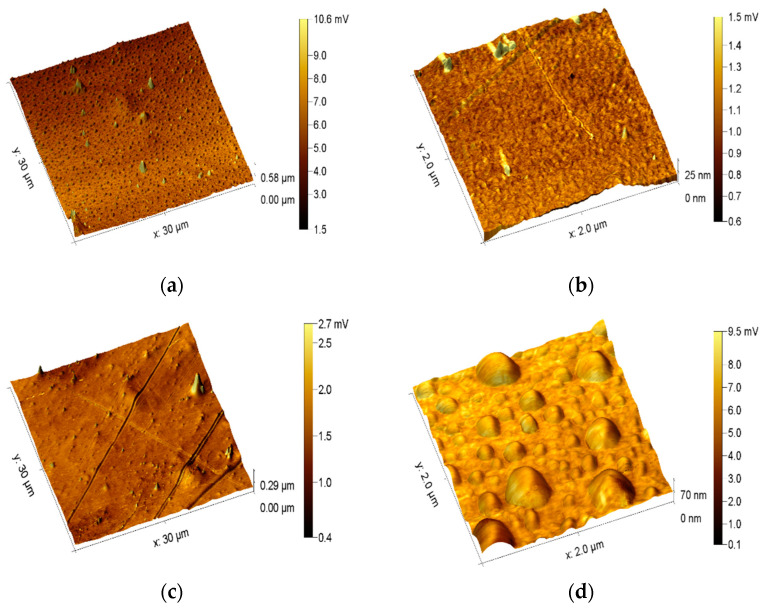

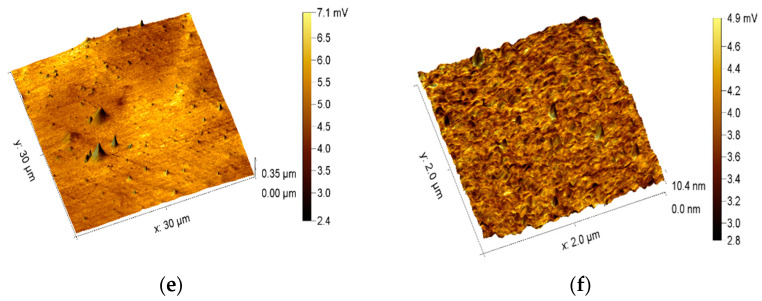
Images of the surface morphology of PET film—atomic force microscopy 3D projections of films topography (the color scale corresponds to the height values): (**a**) PET_control_ (scale 30 μm × 30 μm); (**b**) PET_control_ (scale 2 μm × 2 μm); (**c**) PET_plasma 5min_ (scale 30 μm × 30 μm); (**d**) PET_plasma 5min_ (scale 2 μm × 2 μm); (**e**) PET_plasma 15min_ (scale 30 μm × 30 μm); (**f**) PET_plasma 15min_ (scale 2 μm × 2 μm).

**Figure 2:**
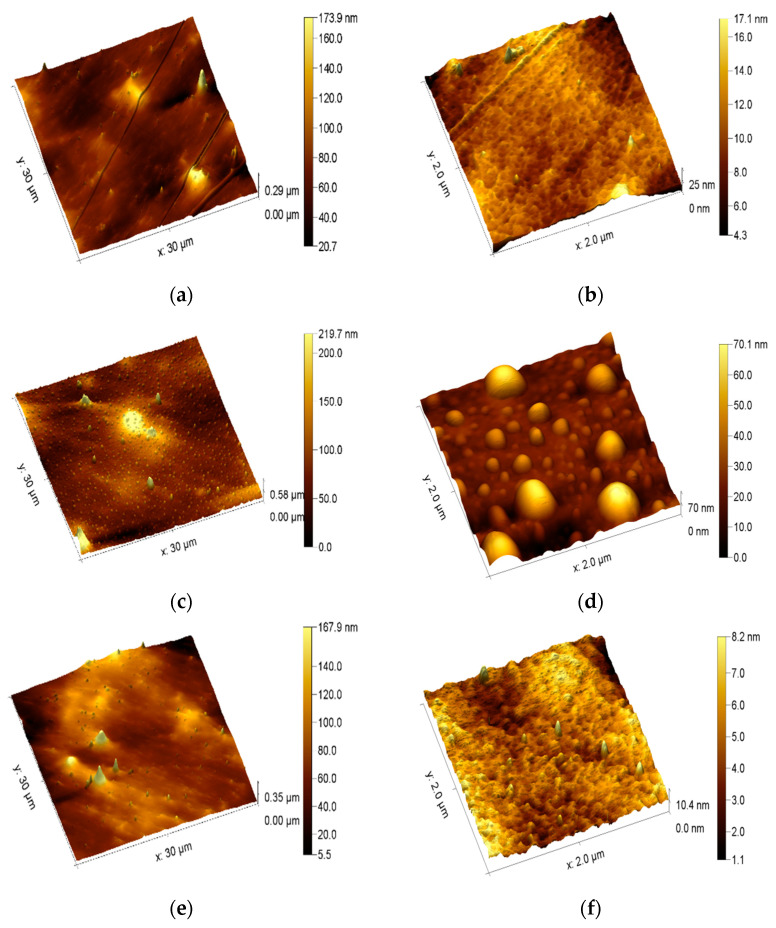
Images of the surface morphology of PET film—lateral force microscopy of PET films (the color scale corresponds to the friction values): (**a**) PET_control_ (scale 30 μm × 30 μm); (**b**) PET_control_ (scale 2 μm × 2 μm); (**c**) PET_plasma 5min_ (scale 30 μm × 30 μm); (**d**) PET_plasma 5min_ (scale 2 μm × 2 μm); (**e**) PET_plasma 15min_ (scale 30 μm × 30 μm); (**f**) PET_plasma 15min_ (scale 2 μm × 2 μm).

Changes in the surface morphology of the PET films were investigated using the AFM technique. As shown in Figure 1, the surface of the control sample is quite smooth. The 3D projections of the AFM and LFM images are presented in Figure 1 and Figure 2, respectively. Some grooves are the residues after the foil manufacturing process. The films modified with atmospheric plasma were characterized by a more developed surface. This effect was very visible for PET treated with plasma for 5 min, where many roundish bubble-like structures were observed. It is interesting that these bubble-like structures were not observed on the surface of the plasma-modified film for 15 min (PET_plasma 15min_).

Thereafter, the pristine PET films and the samples treated with non-thermal plasma were subjected to an aerobic controlled soil biodegradation test. The use of the AFM technique provided interesting information about changes on the surface of the tested polymer. The 3D projections of the AFM topography of the PET films after the biodegradation test are shown in Figure S1 (Supplementary Information). The color scale for Figure S1A represents the topographic features, while Figure S1B shows the LFM images. The color scale corresponds to the friction values. Plasma pretreatment did not seem to affect the differences in the surface morphology of PET films after biodegradation in soil (Figure S1A, Supplementary information). However, some differences can be observed for the nanoroughness changes for 2 µm × 2 µm scans (Figure S1B, Supplementary Information). The bubble-like structures that were seen on the PET film treated with plasma for 5 min before degradation disappeared and left a quite-smooth surface, similar to the surface of other degraded samples.

**Figure 3:**
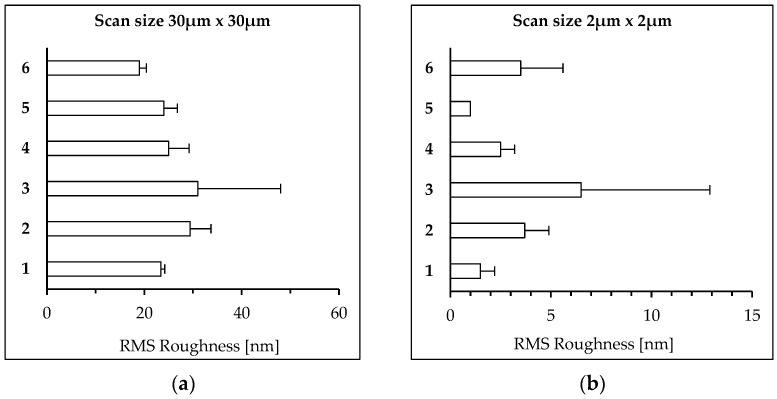
Root mean square (RMS) roughness of the PET foils measured via the AFM: (**a**) roughness in case of 30 µm × 30 µm scans; (**b**) roughness in case of 2 µm × 2 µm scans. 1—PET_control_; 2—PET_biodegraded_; 3—PET_plasma 5min_; 4—PET_plasma 5min + biodegraded_; 5—PET_plasma 15min_; 6—PET_plasma 15min + biodegraded_.

Figure 3 presents the average friction root mean square (RMS) roughness of the PET films measured via AFM. Plasma pretreatment did not seem to affect the differences in the surface morphology of PET films after biodegradation in soil (Figure 3a). However, some differences can be noted for the changes for 2 µm × 2 µm scans (Figure 3b). Quantitative topography analysis has shown that the RMS of biodegraded PET film increases from ~23 to 32 nm, whereas the roughness of the plasma-treated samples decreases from ~43 nm to ~28 nm and from ~26 nm to ~18 nm for PET_plasma 5min + biodegraded_ and PET_plasma 15min + biodegraded_, respectively.

**Figure 4:**
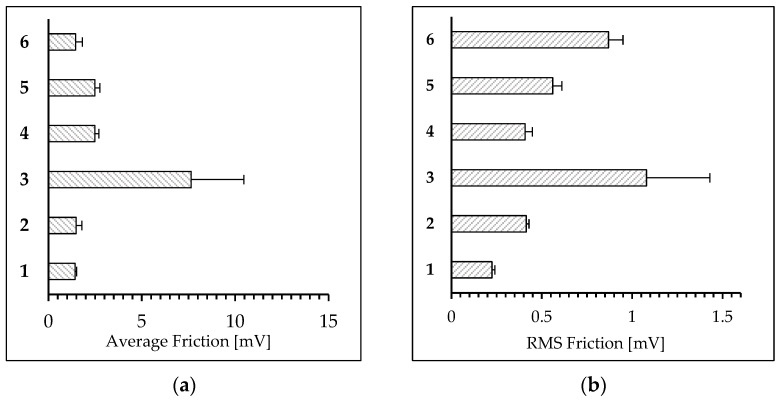
LFM results of surface topography of PET films: (**a**) average friction measured via LFM; (**b**) root mean square (RMS) of friction, denoting the variance of the data. 1—PET_control_; 2—PET_biodegraded_; 3—PET_plasma 5min_; 4—PET_plasma 5min + biodegraded_; 5—PET_plasma 15min_; 6—PET_plasma 15min + biodegraded_.

Figure 4a presents the average friction measured for the 30 µm × 30 µm square via the LFM method, while Figure 4b shows the root mean square (RMS) of friction, which is related to the distribution of friction across the surface.

Both PET_control_ and PET_biodegraded_ exhibited the lowest friction. RMS increased for the PET_biodegraded_ compared to PET_control_. The PET_plasma 15min_ had lower nanofriction than the PET_plasma 5min_. The highest friction was observed for PET_plasma 5min._

### 3.2. Changes in Polymer Film Wettability

**Figure 5:**
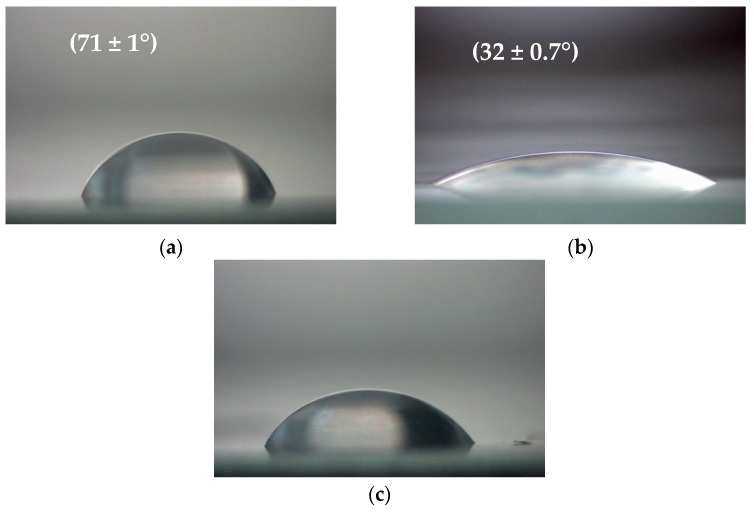
Images of water drops on PET films before (PET_control_) (**a**) and after treatment with the non-thermal plasma for 5 min (PET_plasma 5min_) (**b**), as well as at 15 min (PET_plasma 15min_) (**c**).

The contact angles on the tested polymer were measured immediately after non-thermal plasma treatment, and the obtained results were compared with those of the pristine PET films (i.e., those not exposed to plasma) (Figure 5). It is known that PET is hydrophobic nature [43], and the contact angle of the pristine plastic was 71 ± 1°.

PET films after non-thermal plasma modification showed time-dependent changes in the water contact angles. As can be seen in Figure 5, the PET films showed a large decrease in the water contact angle after 5 min of non-thermal plasma treatment, and this parameter decreased from 71 ± 1° for the pristine film (PET_control_) to 32 ± 0.7° for PET after plasma exposure (PET_plasma 5min_). When the polymer surface was modified with the plasma for 15 min (PET_plasma 15min_), the contact angle changed only slightly and amounted to 64 ± 0.5°.

Changes in the wettability of PET films after biodegradation in soil are shown in Table S2 (see Supplementary Information). When PET_control_ was biodegraded for 60 days (PET_biodegraded_), the contact angle decreased slightly from 71 ± 1° to 64 ± 0.7°, respectively. In the case of the PET film pretreated with the non-thermal plasma for 5 min followed by biodegradation (PET_plasma 5min + biodegraded_), the contact angle was reduced to 56 ± 0.5° compared to the PET_control._ When the polymer surface was modified with plasma for 15 min and then biodegraded in soil (PET_plasma 15min + biodegraded_), the contact angle did not change compared to PET_biodegraded_ (the values of the contact angles were 64 ± 0.7° and 63.5 ± 0.5°, respectively) but decreased compared to the reference sample (PET_control_).

### 3.3. Analysis of Changes in Chemical Properties of PET Films

**Figure 6:**
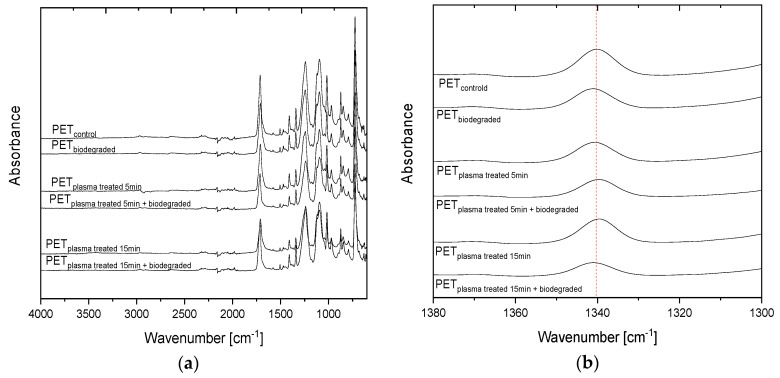
The FTIR-ATR spectra of the reference sample (i.e., PET_control_) and sample after plasma modification and soil incubation (**a**) and enlarged range of 1300–1380 cm^−1^ wavenumbers with the band at 1340 cm^−1^ (**b**).

The FTIR-ATR spectra of the PET samples are combined in Figure 6a. Umamaheswari et al. [44] observed the appearance of three characteristic absorption bands at 1637 cm^−1^ and 1503 cm^−1^ assigned to stretching of the C=C bond in the benzene ring and at 1121 cm^−1^ assigned to stretching of the C–O bond, confirming the formation of the ester group for PET powder after 60 days of incubation in soil. On the contrary, the spectra presented in Figure 6a show significant differences between PET control and PET after plasma modification and soil incubation. The band at 1340 cm^−1^ attributed to vibrations of the *trans* form of the ethylene glycol segment and assigned to the crystalline region [45,46] recorded for PET plasma-modified for 15 min and after degradation in soil exhibits lower intensity than the reference PET_control_ sample (Figure 6b). This observation is consistent with DSC results that confirm a decrease in the degree of PET plasma pretreated for 15 min and after soil incubation compared to reference PET.

**Figure 7:**
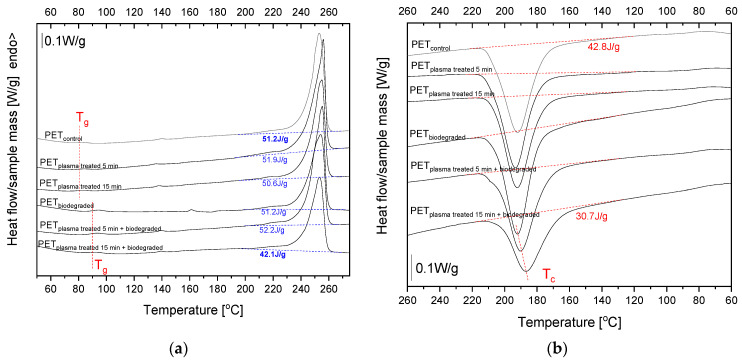
The first heating (**a**) and cooling (**b**) DSC curves of the reference PET_control_ and PET after plasma modification and soil incubation.

To estimate the influence of plasma modification on the thermal properties of PET after aerobic degradation, DSC measurements were performed. The first DSC heating curves are presented in Figure 7, and the estimated thermal parameters are collected in Table 1.

The glass transition temperature (*T_g_*) of the reference PET sample (PET_control_) determined as an inflection point is 79.7 °C. The *T_g_* value is not affected by plasma modification, but an increase in *T_g_* of about 10 °C is evident for PET samples after degradation.

Based on the melting endotherm analysis, the effect of plasma modification on the biodegradation process is clear for PET samples modified for 15 min, as indicated by the lower value of the melting enthalpy (*H_m_*) for PET_plasma 15min + biodegraded_ compared to the reference PET_control_. The degree of crystallinity (*X_c_*) of PET_plasma 15min + biodegraded_ calculated from Δ*H_m_* is 33.7%, while for the reference sample *X_c_*, it is about 7.3% higher.

The results of the GPC analysis of the molecular weight of pristine PET, samples after plasma modification, and films that degraded in soil are summarized in Table S3 (Supplementary Information). As can be seen, the significant differences between the PET films have not been demonstrated.

### 3.4. Analysis of Electrical Changes of PET Films

The effect of the plasma treatment process on the individual electrical parameters of the PET film, i.e., volume and surface resistivity, dielectric loss factor, and electrical strength, was determined during the electrical measurements (Table S4, Supplementary Information). The lowest values of volume and surface resistivities were obtained in the case of PET films after plasma treatment and biodegradation in soil. Control samples (i.e., without plasma and microorganism exposure) exhibited the maximum values of the above parameters. For all cases, the results obtained were of the same order of magnitude, indicating that both the plasma treatment process and the biodegradation in soil do not affect the electrical conductivity of the PET film.

The results of tgδ measurements show that the control sample is characterized by the lowest loss of energy in a dielectric material when placed in an electromagnetic field. Non-thermal plasma or microbial exposure leads to a slight increase in the dielectric loss factor, but the observed differences are at the 10% level.

### 3.5. Analysis of the Effectiveness of Biofilm Development on the Surface of PET Films

The crystal violet assay was used to analyze the effectiveness of biofilm development on the surface of PET films. Colorimetric detection was performed at a wavelength of 595 nm, and the amount of color produced was directly proportional to the number of cells.

**Figure 8:**
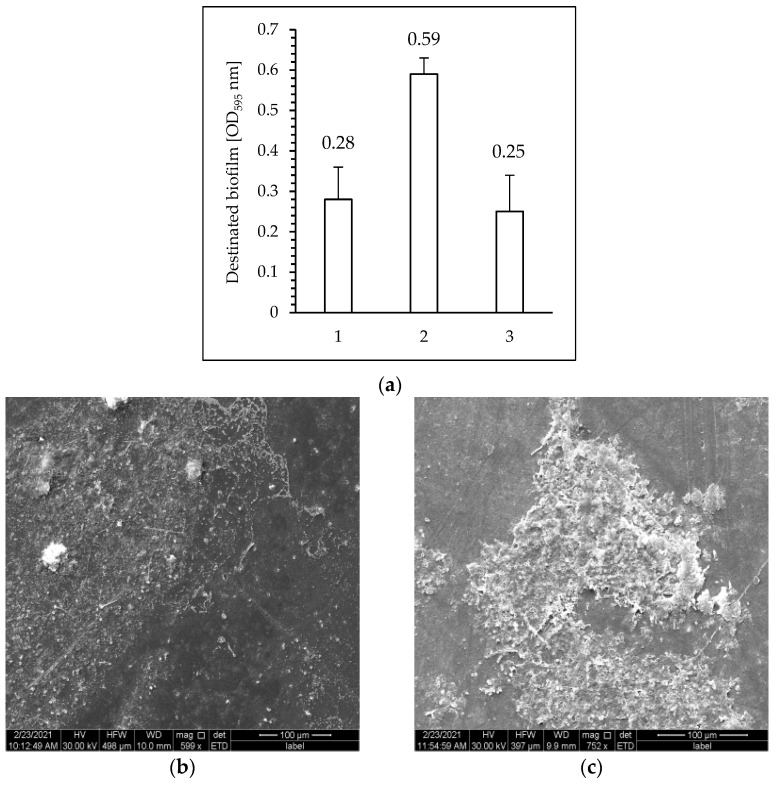
(**a**) Effectiveness of biofilm development on the surface of PET film before plasma treatment (PET_control_) (1); PET film after plasma treatment for 5 min (PET_plasma 5min_) (2); PET film after plasma treatment for 15 min (PET_plasma 15min_) (3). Scanning electron microscopic images of (**b**) PET_control_ and (**c**) PET_plasma 5min_ surfaces.

As can be seen in Figure 8a, a microbial biofilm was formed on the surface of the plasma-treated films as well as on the surface of the PET sample without plasma exposure. The highest biofilm formation efficiency was observed on the PET surface after plasma modification for 5 min (PET_plasma 5min_). The number of cells in the biofilms formed on the surface of PET_control_ and PET modified with plasma for 15 min was similar, but this value was lower than for the sample treated with PET plasma for 5 min.

More effective biofilm formation on the surface of the tested polymer that was pre-treated with plasma (compared to the sample without exposure to plasma) was confirmed via SEM analysis (Figure 8b,c).

### 3.6. The Effect of Plasma Treatment on the Biodegradability of PET Film

The weight loss of the polymer after 60 days of aerobic biodegradation is insignificant and ranges from 0.24 to 0.64% for PET film without plasma pretreatment (PET_biodegraded_) and for plastic after 15 min of plasma exposure (PET_plasma 15min + biodegraded_), respectively (Figure S2, Supplementary Information). Exposure of the PET film to non-thermal plasma for 15 min resulted in the highest mass loss of the polymer during soil degradation (i.e., the mass loss was more than twice as high compared to the samples without plasma exposure and treated with plasma for 5 min).

## 4. Discussion

The paper describes, for the first time, the effect of non-thermal plasma and the subsequent biodegradation in the soil of ultrathin polyethylene terephthalate films on changes in the physicochemical properties of this polymer.

The surface morphology of the PET film was examined after each step of these modifications using the AFM technique. Significant changes in the surface of PET film were revealed after 5 min of plasma treatment (PET_plasma-treated 5min_). These changes were described as bubble-shaped structures. It is accepted that this kind of change in surface morphology is assigned to the etching process [47,48]. It is interesting that these bubble-shaped structures were not observed on the surface of the film plasma-modified for 15 min (PET_plasma 15min_). It should also be noted that after biodegradation in the soil, the bubble-like structures mentioned above disappeared from the surface of the films subjected to plasma pretreatment for 5 min. There were no significant differences between the samples pretreated with plasma and then biodegradable in soil and the samples biodegradable under the same conditions but without plasma modification.

Moreover, all plasma-treated films were shown to have a higher friction compared to the untreated samples, and this phenomenon is usually attributed to oxidization of the surface layer [49]. The roughness of the PET films was tested on two length scales: 30 µm × 30 µm and 2 µm × 2 µm. In the case of the larger scale, the differences between the roughness of the PET film samples were marginal. However, the PET_plasma 5min_ sample was found to be characterized by the highest roughness. The biodegradation of PET films in soil reduced the roughness in all cases to the level of the untreated sample. These differences are more pronounced on the 2 µm × 2 µm scale. Although the roughness is in the range of a few nanometers, it can be seen that the biodegradation increased the roughness of the untreated film. Plasma treatment increased the nanoroughness of PET_plasma 5min_, but this effect was not observed for the PET_plasma 15min_ film. Surprisingly, degradation reduced the roughness of PET_plasma 5min_ but not for the PET_plasma 15min_. Inversely, the latter film had a higher nanoroughness after degradation.

The LFM enables measurement of nanofriction between the AFM tip and the substrate surface. It is related to the formation of a water meniscus between the tip and the substrate. The higher oxidation of the surface leads to a more hydrophilic surface and formation of a thicker structural H_2_O layer, resulting in a higher nanofriction between the tip and the surface. Therefore, higher hydrophilicity leads to higher friction [50].

The increase in friction should be attributed to the oxidation of the surface with plasma treatment. Biodegradation caused degradation of the oxidized layer, resulting in decrease in friction. The biodegradation of PET_plasma 5min+ biodegraded_ maintained higher friction than the degradation of PET_plasma 15min +biodegraded._ In the case of the latter sample, the friction was similar to that of the untreated surface. However, a high RMS was observed for this sample. It can be assumed that since most of the oxidized layer deteriorated, some areas retained their functionalization.

It is worth emphasizing that the plasma device used at the given power conditions during the 5-min material processing did not cause an increase in temperature inside the device; the temperature of the dielectric barrier (i.e., ceramic layer) and the high-voltage electrode was below 40 °C, which excludes the possibility of thermal damage to the polymer film. However, after the process, some tarnished areas of the polymer film could be observed, indicating the appearance of so-called hotspots as a result of numerous microdischarges.

Our studies also showed that DBD plasma influenced the contact angle of water on the PET film surface in a time-dependent manner. Plasma treatment (plasma dose of 22.5 Jcm^2^) significantly decreased water contact angle value, while plasma treatment extended to 15 min (plasma dose of 67.5 Jcm^2^) did not change the wettability of the PET surface. It should be noted that after the biodegradation of this polymer in the soil, the value of the contact angle decreased compared to that of the pristine film (PET_control_). The highest decrease in the contact angle value was observed for the PET film pretreated with plasma for 5 min, followed by the soil biodegradation test. The decrease in contact angle is supposed to be connected to changes in surface chemistry and indicates an increase in surface polarity. The interaction of PET films with plasma results in the break of the –C–O– bond in ester groups in the polymer chain [51]. The shorter plasma treatment time (i.e., 5 min) resulted in a higher decrease in the contact angle for the tested polymer. Based on the data from the literature, we believe that the activation of the surface was a consequence of functionalization with oxygen functional groups (probably OH radicals) [52]. According to the Cassie–Baxter models, not only surface chemistry but also surface roughness are the primary factors determining the behavior of liquid droplets on a solid surface [52].

Interesting changes in the chemical properties of PET samples after plasma pretreatment and biodegradation in soil were revealed via FTIR-ATR spectra. The decrease in band intensity at 1340 cm^−1^ observed for plasma-modified PET for 15 min and after degradation in soil suggested a decrease in the degree of crystallinity of this polymer. This phenomenon was confirmed by the DSC results. The decrease in the content of the crystalline phase of the PET after soil degradation has not been described so far. The result to be expected was the result of an increase in the degree of crystallinity resulting from the typically observed deterioration mechanism occurring with the degradation of the amorphous phase in the first stage of the process. Since the observed decrease in *X_c_* occurs only for the PET film pretreated with plasma for 15 min, this allows us to conclude that the plasma modification induced such biodeterioration, resulting in the destruction of the PET crystalline structure. This effect does not occur for PET film modified with plasma over a shorter period (i.e., PET_plasma 5min + biodegraded_). The observed effect is uncommon because, in the case of biodegradable polyesters, degradation starts in the amorphous regions, where the density of polymeric chains is lower, facilitating the diffusion of water and the cleavage of ester bonds. Therefore, an increase in the degree of crystallinity is usually observed [53]. Moreover, during the experiment, PET samples were kept at a temperature below *T_g_*, so the material was in a glassy state. In this state, the reorganization of the molecular structure seems to be practically impossible. The polymer chains in the amorphous part are frozen, whereas the rest are blocked in the crystalline phase. It can be concluded from the cooling curves that for samples after degradation, melt crystallization is restricted for PET modified with plasma by 15 min. The peak temperature of crystallization (*T_c_*) is shifted to lower values (about 6.2 °C compared to reference PET). The phenomenon of a decrease in the degree of the crystallinity of PET film after a 15-min plasma modification and then aerobic biodegradation in the soil requires explanation. Research in this area is already underway.

Studies on changes in the number and average molecular weights of PET samples, carried out via the GPC technique, did not reveal significant differences between pristine PET and samples after plasma pretreatment or after aerobic biodegradation in soil. It is known that under conditions where the degradation temperature is lower than the *T_g_*, water cannot easily diffuse into the material, and its uptake is very low. Thus, hydrolysis in whole volume is very difficult. In the case of our samples, the degradation temperature was far below *T_g_*, and only superficial hydrolysis occurred. Therefore, it can be difficult to observe significant changes in the degraded molecular weight of the polymer under soil conditions. Moreover, superficial degradation also causes changes in the surface morphology of the degraded samples. In our experiments, these effects were observed but at a relatively low level.

As mentioned above, no significant differences in electrical properties were observed between pristine PET film and samples after plasma modification and biodegradation in soil. Only higher standard deviations of the electric strength values were recorded for the samples modified with plasma or those biodegradable in soil compared to the control sample. This effect can be explained by an increase in the number of surface defects of the sample due to its exposure to plasma or microorganisms. On the basis of the electrical test results, it can be concluded that the influence of plasma and soil biodegradation is negligible on *_v_*, *_s_*, tg*δ*, and *E_f_*. Note that the determined parameters (excluding the surface resistivity) refer to the bulk properties of the material; therefore, it can be assumed that both processes (i.e., plasma treatment and biodegradation) are mainly relevant to the surface parts of the material.

Biofilms are assemblages of single or multiple populations that are attached to abiotic or biotic surfaces through extracellular polymeric substances. During biofilm development, primary colonizers (i.e., pioneering species) attach first, and when biofilm conditions change, secondary colonizers are able to attach themselves to already-established organisms, ultimately leading to a stable community [53]. Microorganisms also create biofilms at the sites where material degradation occurs. One of the main factors influencing the formation of biofilms on the surface of various materials is the process of adhesion to the surface (co-adhesion) and adhesion between cells of various types and species (coaggregation) [54]. It is widely believed that the hydrophobicity of the surface and the roughness of the materials play an important role in the initial adhesion of the microbes and the subsequent biofilm formation of biofilms [55]. However, there is still disagreement about how the hydrophobicity and roughness of biomaterials determine the development [56]. For example, De-la-Pinta et al. [57] demonstrated that staphylococcal biofilms were less common on surfaces with medium roughness (i.e., below bacterial diameter (0.38 µm)) than on surfaces with higher roughness (above 0.51 µm). These authors believed that the reduction in bacterial adhesion was associated with a reduction in the ability of the contact surface to interact with microorganisms.

In the present study, it was found that the efficiency of biofilm formation depended on the increase in surface polarity. It should be taken into account that in our experiments, all samples were relatively smooth. As mentioned previously, the RMS was approximately 23 nm for pristine PET, whereas for the 5 min and 15 min plasma-treated samples, it was ~43 nm and ~26 nm, respectively. However, these data were obtained for 30 µm × 30 µm. For smaller ones—that is, 2 µm × 2 µm—the RMS values were even lower (i.e., in the range of 1–11 nm). Therefore, it seems that surface roughness was not the crucial factor that affected the bacterial adhesion process. These results are in agreement with the previous observations of some authors; for example, Seddiki et al. [58] showed that surface modifications on the nanoscale can also lead to a decrease in biofilm formation due to the relatively large size of a common bacteria cells relative to the surface indents. It has already been shown that in addition to facilitating attachment, the roughness of the surface can also lead to physiological changes in the bacteria that colonize it, such as the loss of flagella and the accompanying reduction in mobility [59]. It is generally accepted that the formation of a biofilm on the surface of polymers is a prerequisite for their biodegradation [60]. Biofilm formation can alter the physicochemical properties of plastic, including changes in functional groups, hydrophobicity/hydrophilicity, crystallinity, surface, morphology, and molecular weight distribution [61].

Poly(ethylene terephthalate) have been recognized as chemically inactive and highly resistant to microbial exposure. It is believed that the main cause of the poor biodegradability of PET is the minimally reactive C–C bonds in the backbone [62]. Moreover, polymer chain flexibility, crystallinity, and surface hydrophobicity have been identified as unfavorable for the biodegradation of this polymer [63].

Generally, to determine the biodegradability of plastic, a polymer sample is incubated with soil, compost, or activated sludge under strict conditions, and the degradation rate is compared with a biodegradable reference sample, such as cellulose [64]. A polymer can be classified as “biodegradable” when more than 60–70% of the sample has degraded within 6 months. An important parameter indicating biodegradation efficiency is weight loss due to biochemical processes that occur in the environment.

Our study on the weight loss of PET film after 60 days of aerobic biodegradation in soil showed that pretreatment with plasma for 15 min affects the biodegradation efficiency of this polymer (see Supplementary Information). Weight loss was negligible (it did not exceed 0.64%), but it is worth noting that the samples pretreated with plasma for 15 min lost almost three times the weight of those without plasma modification.

Poly(ethylene terephthalate) monomers are linked by ester bonds, which theoretically can be hydrolyzed by naturally occurring hydrolytic enzymes. To date, many hydrolytic enzymes have been identified, but only a few reports have shown their ability to degrade PET. Recently, a rod-shaped Gram-negative bacterium has been found to be a new species of the genus *Ideonella* (*I. sakaiensis* 201-F6), capable of metabolizing amorphous PET [62]. Sequencing of the *I. sakaiensis* genome allowed for the identification of an enzyme with PET hydrolyzing properties. This enzyme was designated as PET hydrolase (PETase), showing the highest catalytic preference for PET at room temperature [63]. Janczak et al. [65] observed that the combination of bacteria and fungi with some plant species (mainly *S. plymuthica)* significantly increased the microbial population in the soil and the degradation efficiency of PET films.

## 5. Conclusions

The use of non-thermal plasma to modify the properties of PET films may result in an increase in their roughness and wettability, as well as increased biofilm formation on the material surface. The main novelty of the work is the fact that the combined action of two factors (i.e., physical and biological) led to a reduction in the content of the crystalline phase in the tested polymeric material. The changes in the crystalline structure of PET film caused by this operation require more attention. This research is currently underway.

## Figures and Tables

**Table 1:** Thermal parameters of the studied PET films.

Sample	1st Heating				Cooling		
	*T_g_* [°C]	*T_m_* [°C]	Δ*H_m_* [J/g]	*X_c_* [%]	*T_c_* [°C]	Δ*H_c_* [J/g]	*T_g_* [°C]
PET_control_	79.7	253.1	51.2	41.0	191.9	42.8	84.5
PET_plasma 5min_	80.3	256.0	51.9	41.5	193.4	42.8	81.0
PET_plasma 15min_	79.3	254.9	50.6	40.5	192.4	41.0	81.7
PET_biodegraded_	90.7	255.2	51.2	41.0	192.2	43.3	82.0
PET_plasma 5min + biodegraded_	89.8	254.1	52.2	41.8	190.2	40.4	82.4
PET_plasma 15min + biodegraded_	90.2	253.3	42.1	33.7	186.7	30.7	88.8

## Data Availability

Not applicable.

## Supplementary Materials

The following supporting information can be downloaded at https://www.mdpi.com/article/10.3390/polym15214289/s1: Table S1: Chemical and physical analysis of soil samples used in experiments; Figure S1: Surface topography of PET films: A—Atomic Force Microscopy 3D projections of the biodegraded films topography; the color scale corresponds to the height values. B—Lateral Force Microscopy of PET films. Images shows 3D projections of the topography, the color scale corresponds to the Friction values; Table S2: The water contact angle of the PET films after biodegradation in soil; Table S3: Number and weight average molecular weights of PET samples; Table S4: Electrical parameters of the PET films; Figure S2: The percentage weight reduction of PET film after 60 days of biodegradation: PET_biodegraded_ (without plasma treatment) (1); PET_plasma 5 min+biodegraded_ (2); PET_plasma 15 min+biodegraded_ (3); the result is given as the mean of triplicate.
