# Supplementary material for: On the Effect of Non-Thermal Atmospheric Pressure Plasma Treatment on the Properties of PET Film
Source: Polymers (Basel). 2023 Oct 31;15(21):4289. doi: 10.3390/polym15214289 (PMC10650147; doi:10.3390/polym15214289)
Supplement: Supplementary file 1 [file polymers-15-04289-s001.zip › polymers-2666219-supplementary.pdf]

Supplementary Information

On the Effect of Non-Thermal Atmospheric Pressure Plasma Treatment on the Properties of PET Film

Irena Maliszewska[1*], Małgorzata Gazińska[2*], Maciej Łojkowski[3,4], Emilia Choińska[3], Daria Nowinski[1], Tomasz Czapka[5], Wojciech Święszkowski[3]

[1]Department of Organic and Medicinal Chemistry, Faculty of Chemistry, Wrocław University of Science and Technology, Poland; irena.helena.maliszewska@pwr.edu.pl; daria.kocek@pwr.edu.pl
[2]Department of Polymer Engineering and Technology, Faculty of Chemistry, Wrocław University of Science and Technology, Poland; malgorzata.gazinska@pwr.edu.pl
[3]Faculty of Materials Science and Engineering, Warsaw University of Technology, Poland; maciek.lojkowski@pw.edu.pl; emilia.choinska@pw.edu.pl; wojciech.swieszkowski@pw.edu.pl
[4]Centre for Advanced Materials and Technology CEZAMAT, Warsaw University of Technology, Poland; maciek.lojkowski@pw.edu.pl
[5]Department of Electrical Engineering Fundamentals, Faculty of Elecrical Engeenering, Wrocław University of Science and Technology, Poland; tomasz.czapka@pwr.edu.pl

Correspondence: irena.helena.maliszewska@pwr.edu.pl

Table S1. Chemical and physical analysis of soil samples used in experiments

Parameter	Value
TOC, $mgkg^{-1}$	16.48
TKN, $mgkg^{-1}$	3.41
pH	7.35
C:N	11.2

A

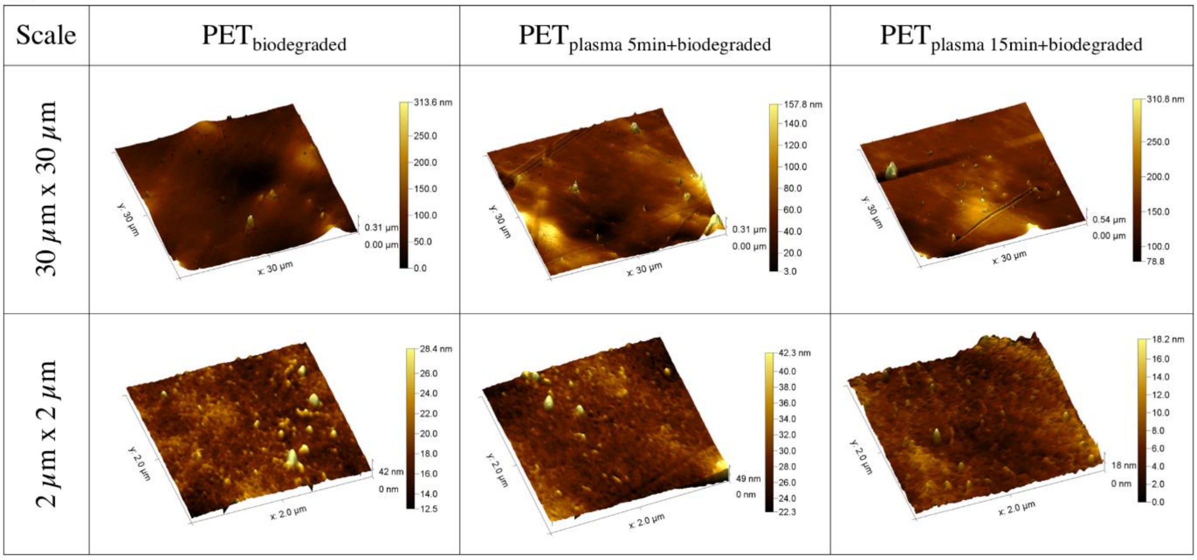

B

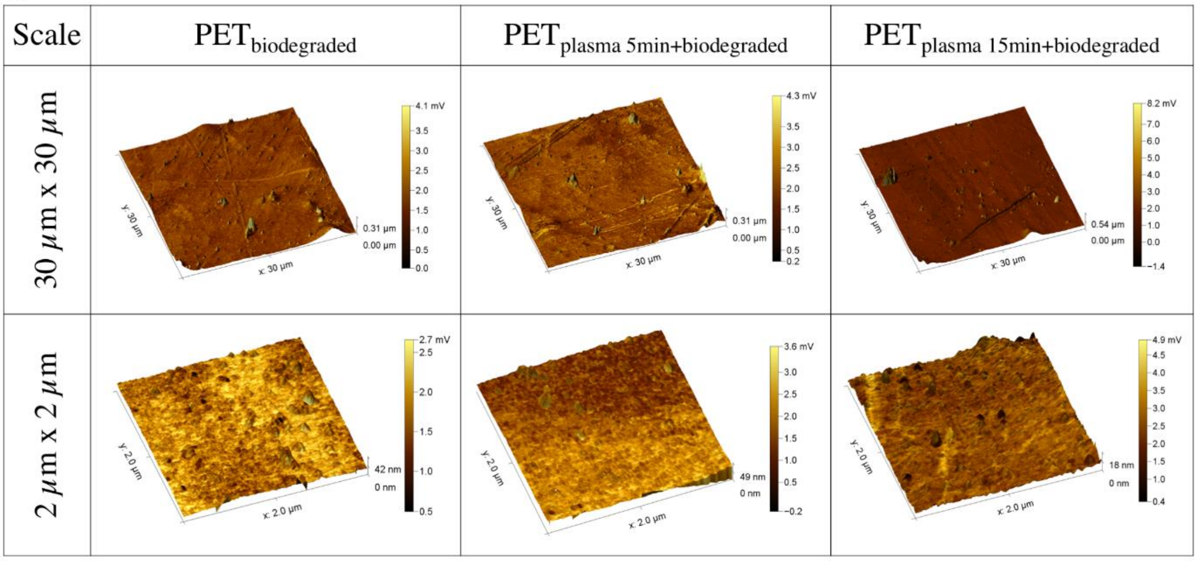

Figure S1. Surface topography of PET films: A - Atomic Force Microscopy 3D projections of the biodegraded films topography; the color scale corresponds to the height values. B - Lateral Force Microscopy of PET films. Images shows 3D projections of the topography, the color scale corresponds to the Friction values.

Table S2. The water contact angle of the PET films after biodegradation in soil

Sample	θ
$PET_{biodegraded}$	64±0.7
$PET_{plasma\ 5min+biodegraded}$	56±0.5
$PET_{plasma\ 15min+biodegraded}$	63.5±0.5

Table S3. Number and weight average molecular weights of PET samples

Sample	Mn (kDa)	Mw (kDa)	PDI (kDa)
$PET_{control}$	23.7	47.3	2.00
$PET_{biodegraded}$	23.4	47.4	2.03
$PET_{plasma\ 5\ min}$	24,1	47,4	1,97
$PET_{plasma\ 5\ min+biodegraded}$	23,2	47,1	2,03
$PET_{plasma\ 15\ min}$	23.3	47.1	2.02
$PET_{plasma\ 15min+biodegraded}$	23.8	47.0	1.97

Table S4. Electrical parameters of the PET films

**Film tested**	**Parameter**			
	**Volume resistivity ρ_v, Ωm**	**Surface resistivity ρ_s, Ωm**	**Dielectric loss factor tgδ, -**	**Electrical strength, E_f, V/m**
$PET_{control}$	$(7.1±0.1)·10^{15}$	$(3.0±0.1)·10^{14}$	$(1.9±0.2)·10^{-3}$	$(106±10)·10^{6}$
$PET_{plasma15\ min}$	$(5.4±0.2)·10^{15}$	$(2.7±0.2)·10^{14}$	$(2.2±0.2)·10^{-3}$	$(108±15)·10^{6}$
$PET_{biodegraded}$	$(6.8±0.2)·10^{15}$	$(2.6±0.2)·10^{14}$	$(2.4±0.3)·10^{-3}$	$(101±15)·10^{6}$
$PET_{plasma\ 15min+biodegraded}$	$(2.3±0.2)·10^{15}$	$(2.4±0.2)·10^{14}$	$(2.4±0.3)·10^{-3}$	$(109±15)·10^{6}$

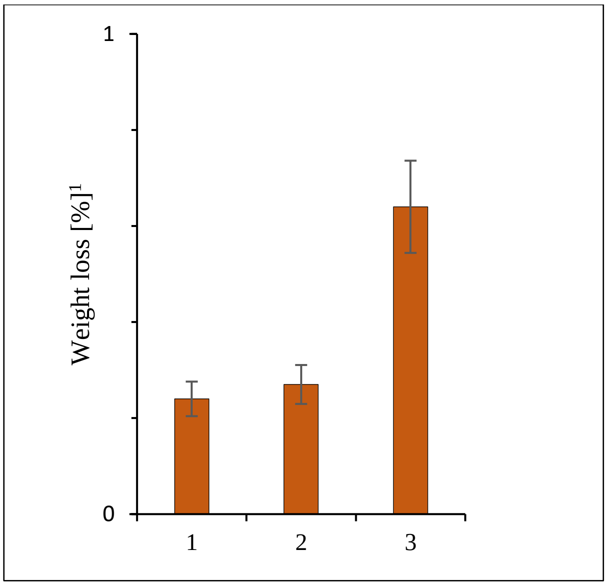

Figure S2. The percentage weight reduction of PET film after 60 days of biodegradation: $PET_{biodegraded}$ (without plasma treatment) **(1)**; $PET_{plasma\ 5\ min+biodegraded}$ **(2)**; $PET_{plasma\ 15\ min+biodegraded}$ **(3)**; the result is given as the mean of triplicate
